# Autoantibodies against oncogenic ERG protein in prostate cancer: potential use in diagnosis and prognosis in a panel with C-MYC, AMACR and HERV-K Gag

**DOI:** 10.18632/genesandcancer.126

**Published:** 2016-11

**Authors:** Anshu Rastogi, Amina Ali, Shyh-Han Tan, Sreedatta Banerjee, Yongmei Chen, Jennifer Cullen, Charles P. Xavier, Ahmed A. Mohamed, Lakshmi Ravindranath, Jigisha Srivastav, Denise Young, Isabell A. Sesterhenn, Jacob Kagan, Sudhir Srivastava, David G. McLeod, Inger L. Rosner, Gyorgy Petrovics, Albert Dobi, Shiv Srivastava, Alagarsamy Srinivasan

**Affiliations:** ^1^ Center for Prostate Disease Research, Department of Surgery, Uniformed Services University of the Health Sciences, Bethesda, MD, USA; ^2^ Urology Service, Department of Surgery, Walter Reed National Military Medical Center, Bethesda, MD, USA; ^3^ The Joint Pathology Center, Silver Spring, MD, USA; ^4^ Cancer Biomarkers Research Group, Division of Cancer Prevention, National Cancer Institute, Bethesda, MD, USA

**Keywords:** prostate cancer, diagnosis, autoantibody, panel, ERG

## Abstract

Overdiagnosis and overtreatment of prostate cancer (CaP) is attributable to widespread reliance on PSA screening in the US. This has prompted us and others to search for improved biomarkers for CaP, to facilitate early detection and disease stratification. In this regard, autoantibodies (AAbs) against tumor antigens could serve as potential candidates for diagnosis and prognosis of CaP. Towards this, our goals were: i) To investigate whether AAbs against ERG oncoprotein (overexpressed in 25-50% of Caucasian American and African American CaP) are present in the sera of CaP patients; ii) To evaluate an AAb panel to enhance CaP detection. The results using an enzyme-linked immunosorbent assay (ELISA) showed that anti-ERG AAbs are present in a significantly higher proportion in the sera of CaP patients compared to healthy controls (*p* = 0.0001). Furthermore, a panel of AAbs against ERG, AMACR and human endogenous retrovirus-K Gag successfully differentiated CaP patient sera from healthy controls (AUC = 0.791). These results demonstrate for the first time that anti-ERG AAbs are present in the sera of CaP patients. In addition, the data also suggest that AAbs against ERG together with AMACR and HERV-K Gag may be a useful panel of biomarkers for diagnosis and prognosis of CaP.

## INTRODUCTION

Prostate cancer (CaP) is a prevalent disease among US men and accounts for a total of an estimated 180,890 cases diagnosed in 2016 with 26,120 deaths [1]. The assays currently used in the diagnosis of CaP include screening for prostate-specific antigen (PSA), digital rectal examination (DRE) and biopsy [2, 3]. However, convincing evidence demonstrates that the PSA test often produces false-positive results: approximately 80% of positive results are false when using a cutoff between 2.5 and 4.0 μg/L [4]. This leads to a substantial number of men which are over-diagnosed, resulting in over-treatment for CaP [5], therefore the U.S. Preventative Services Task Force recently recommended against PSA-based screening for CaP [6]. Thus, it has become an area of high priority in the field of CaP research to find new biomarkers and develop assays, which can provide a better diagnostic scenario for patients.

While it is well accepted that early detection of CaP can result in over-treatment for some patients, it can be beneficial for patients with progressive disease that will become refractory to treatments. As a multifocal heterogeneous disease, CaP poses at least two challenges from the diagnostic perspective: i) Screening methods for the detection of early disease (e.g. PSA, RT-PCR, and IHC); ii) Biomarkers that can differentiate indolent from aggressive tumors early in the continuum of cancer care. In general, CaP and other cancers can greatly benefit from minimally invasive blood-based screening tests in comparison to invasive approaches for diagnosis. The assays currently used share several disadvantages which include: limited sensitivity and/or specificity, impractical clinical implementation, and high costs [7-9]. Recently, two mRNA-based urine assays have become available on the market using PCA3 and a combination of ERG, PCA3, and SPDEF, by GenProbe and Exosome Diagnostics, respectively, which are being used to facilitate decisions about the need for biopsies in patients [10]. Prognostic assays that detect the expression of cellular genes using samples derived from needle biopsies or radical prostatectomy specimens are currently available, such as Prolaris (Myriad Genetics) [11], Oncotype DX® (Genomic Health) [12-14], and Decipher (Genome DX). Although, these assays contribute to improved risk assessment, the overall cost is much higher. This scenario has led investigators to consider alternative strategies such as measuring tumor antigens and autoantibodies (AAbs) in body fluids such as serum/plasma [15-18]. It should be mentioned that AAbs, in comparison to antigens, are not only stable but their response is likely to be generated early on in the course of disease [19]. AAbs represent humoral immune responses of the body against immunogenic tumor-associated antigens (TAAs) highly expressed in tumor cells, and are therefore considered as reporters of the host immune system [19]. Thus, AAbs may be generated well before the overt symptoms of the disease appear [20]. Hence, a test based on AAbs is likely to detect cancer at an early stage. This view is amply supported by data in many different cancers [21-29], with reports of a direct correlation between serum anti-p53 antibodies and p53 overexpression in the corresponding tissue as an example [30]. It should be noted that AAbs to a panel of six or seven tumor antigens (p53, c-MYC, Her-2, NY-ESO-1, MUC1, CAGE and GBU4-5) have been shown to successfully detect lung cancer [31-35] and a similar panel approach is also under consideration for breast cancer [36-39]. Recently, Mintz et al. [40] reported that AAbs against fetuin-A were noted in sera years before the onset of metastatic prostate disease. These findings make the case that AAbs could be used as potential biomarkers for early detection and also as prognostic markers associated with progression of the disease.

AAbs to TAAs have been identified using lysates of established tumor cell lines and tumor cells as a source of antigens for screening against sera. Peptide and phage-display libraries have also been used to identify peptides binding to patient derived sera, ultimately leading to the identification of the candidate protein responsible for the induction of the humoral immune response [41-51]. Studies conducted by our laboratory and others identified the frequent *ERG* oncogene overexpression in CaP cells [52-55]. Independently, Tomlins et al. [56] reported that recurrent gene fusions result in higher expression of ERG in CaP. The predominant gene fusion involved the androgen inducible *TMPRSS2* promoter with *ERG*, a member of the ETS family of transcription factors [8, 57-59]. Interestingly, analysis of the frequency of recurrent gene fusions of *ERG* among diverse racial/ethnic groups has shown varying levels of expression in CaP patients [60-63]. Specifically, Caucasian Americans (CA) have shown to harbor this gene fusion in around 50 % of CaP cases, while African Americans (AA) have shown a lower level of roughly 20-30% of CaP patients. Regarding other racial/ethnic groups, ERG prevalence has been shown at variable levels [9, 64-66]. As a result, there have been efforts to develop two new tests for the detection of CaP using this gene fusion. The first is based on utilizing reverse transcription-polymerase chain reaction (RT-PCR) for the detection of the *TMPRSS2-ERG* gene fusion at the mRNA level [67]. The second involves the testing of biopsied tissue from the prostate gland to assess the expression of ERG oncoprotein by immunohistochemistry (IHC) for stratification of cancer status [62]. Recently, the CPDR laboratory and others have developed highly specific monoclonal antibodies against ERG oncoprotein which have been successfully utilized in IHC studies [7, 68, 69].

In this study, a direct approach was utilized based on CaP biology. Considering the presence of *TMPRSS2-ERG* fusion gene and demonstration of overexpression of ERG protein in a high percentage of CaP patients by IHC [30, 61], we hypothesized that ERG may lead to the induction of anti-ERG AAbs. This study aims to determine the following: i) Whether AAbs against ERG are present in the sera of CaP patients; ii) Whether a multiplex AAb panel containing ERG, AMACR, C-MYC, and human endogenous retrovirus-K (HERV-K) Gag improves the detection of CaP. The results presented here demonstrate that AAbs against ERG protein are present in the sera of CaP patients indicating that ERG is a highly immunogenic protein. Further, the results indicate that a panel of AAbs comprising ERG, C-MYC, AMACR and HERV-K Gag prove to be useful for detecting true CaP cases from controls.

## RESULTS

### Development and optimization of ELISA for the detection of AAbs against ERG oncoprotein

**Figure 1:**
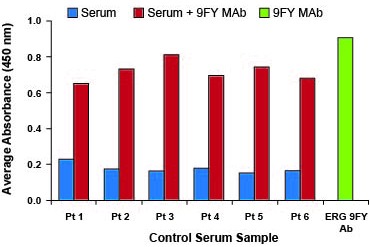
Antibody detection by ELISA Healthy donor control sera (designated as C1-C6), negative for ERG AAbs, were spiked with 10 ng/ml of ERG MAb 9FY and assayed for detection. Positive reactivities indicate that recombinant ERG protein coated on microtiter wells serves as a suitable substrate for AAb detection. ERG MAb 9FY, tested alone as a positive control, is shown in green. Control sera and control sera spiked with monoclonal antibody are represented by blue and red, respectively.

Currently, there is no commercially available diagnostic test for assessing the presence of AAbs against ERG protein in the sera of CaP patients. For this reason, we have developed an in-house assay based on ELISA. For all experiments, 50 ng of recombinant full length ERG protein or 500 ng of peptide were used for coating microtiter wells, based on our previously published work [70]. It has been shown that the ERG 9FY mouse monoclonal antibody (MAb) and the Epitomics ERG rabbit MAb (# 5115) recognize epitopes located at the N-and C-terminal regions of the ERG protein, respectively [7, 69]. The reactivity of each antibody to recombinant ERG protein coated in microtiter wells suggested that the protein is likely accessible to AAbs targeting multiple epitopes, present in patient sera. Initially, we optimized the assay by carrying out spike-in ELISA experiments, in order to mimic analysis involving AAbs in patient sera. Specifically, we selected six candidate human sera from healthy controls and spiked in the ERG MAb 9FY (10 ng/ml). As expected, ELISA results showed high absorbance values both in sera spiked with ERG MAb 9FY (Figure 1) and 9FY alone. Similar results were also noted with Epitomics antibody targeting an epitope at the C-terminal region of ERG protein. In addition, we also observed positive reactivities of sera spiked with ERG MAb 9FY using a peptide representing an epitope located at N-terminal region of the ERG protein as a substrate (data not shown). These results indicated that both recombinant ERG protein and peptides are suitable substrates for detecting AAbs against ERG present in the sera of CaP patients.

### Analysis of sera from CaP patients for the presence of AAbs against ERG

The premise for our studies was that the host immune system produces AAbs against selected tumor antigens which can be detected in blood derived products such as sera and plasma. Based on a high level of ERG protein expression due to genetic alterations in CaP tissues, we questioned, whether anti-ERG AAbs are induced in CaP patients. Earlier studies from our laboratory and others have reported that the extent of genetic rearrangement involving *ERG* and *TMPRSS2* is higher in CA than AA and other ethnic group patients [64]. Taking this into account, for this exploratory study, we have examined sera from age-matched CA CaP patients and healthy controls. The comparison of clinical variables in our cohort is shown in Table 1.

**Table 1:** Descriptive statistics between case and control groups

Variable	Control (N=37)	Case (N=93)	p-value
Age(year)			
Median (range)	55.0(41.0-86.0)	58.8(41.3-76.8)	0.2099
PSA (ng/ml)			
Median (range)	0.94(0.25-1.95)	5.04(0.88-33.00)	<.0001

**Figure 2:**
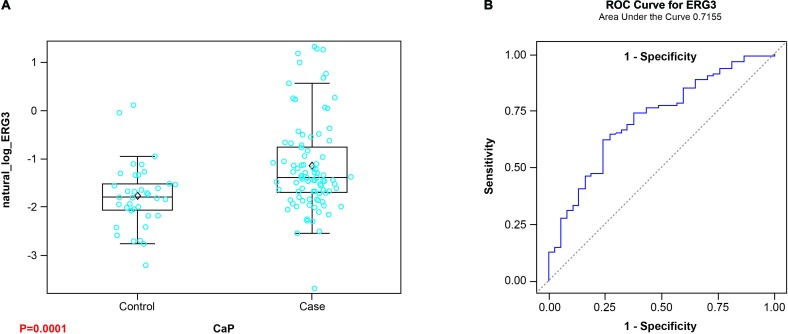
Detection of ERG AAbs in CaP patient sera **A.** Box plots displaying the detection of AAbs against ERG protein in patient sera (p = 0.0001) for CaP Cases vs. Healthy Controls. **B.** Receiver operator characteristic analysis for ERG (AUC = 0.716).

The testing of the sera from CaP patients was carried out at 1:50 dilution by ELISA. The results showed reactivity of CaP patient sera towards ERG protein, indicating the presence of ERG AAbs in the sera of patients. Based on the analysis of the sera, CaP patients exhibited high, intermediate, and low levels of reactivities towards ERG protein (Figure 2A). Sera from healthy controls showed a lower positivity for ERG AAbs. Receiver operating characteristic (ROC) curve analysis for ERG showed an area under the curve (AUC) of 0.716 (Figure 2B). Since it has been suggested that tumor antigens are released from cells either actively or through lysis of tumor cells, we considered the possibility that ERG protein may also be present in patient sera. Hence, it is likely that the quantification of ERG AAbs in patient sera might be affected by the presence of ERG antigen due to immune complex formation. To rule out this possibility, control and CaP patient sera were tested for the presence of ERG antigen for a selected number of patients (based on a range of AAb reactivity) by using a sandwich ELISA, described previously by our laboratory [71]. The results showed that there is no detectable ERG antigen in CaP patient sera by ELISA (data not shown). Together these results indicate that AAb data are total values, and that AAbs against oncogenic ERG are produced and detected only in a subset of CaP patients with varying frequencies and levels.

### Analysis of the specificity of anti-ERG AAbs in the sera of CaP patients

The specificity of the anti-ERG AAbs was determined by multiple approaches. These include: i) Serial dilution of selected patient sera for assessing AAb reactivities towards ERG; ii) Serial dilution of purified total IgG from the CaP patient sera, positive for AAbs, for evaluation of reactivities towards ERG; iii) Competitive ELISA studies using purified IgG from CaP patients; iv) Assessment of the reactivity of purified IgG from patient sera towards ERG protein expressed in VCaP cells using immunofluorescence assays.

### Serial dilution of the patient sera for assessing reactivities towards ERG

**Figure 3:**
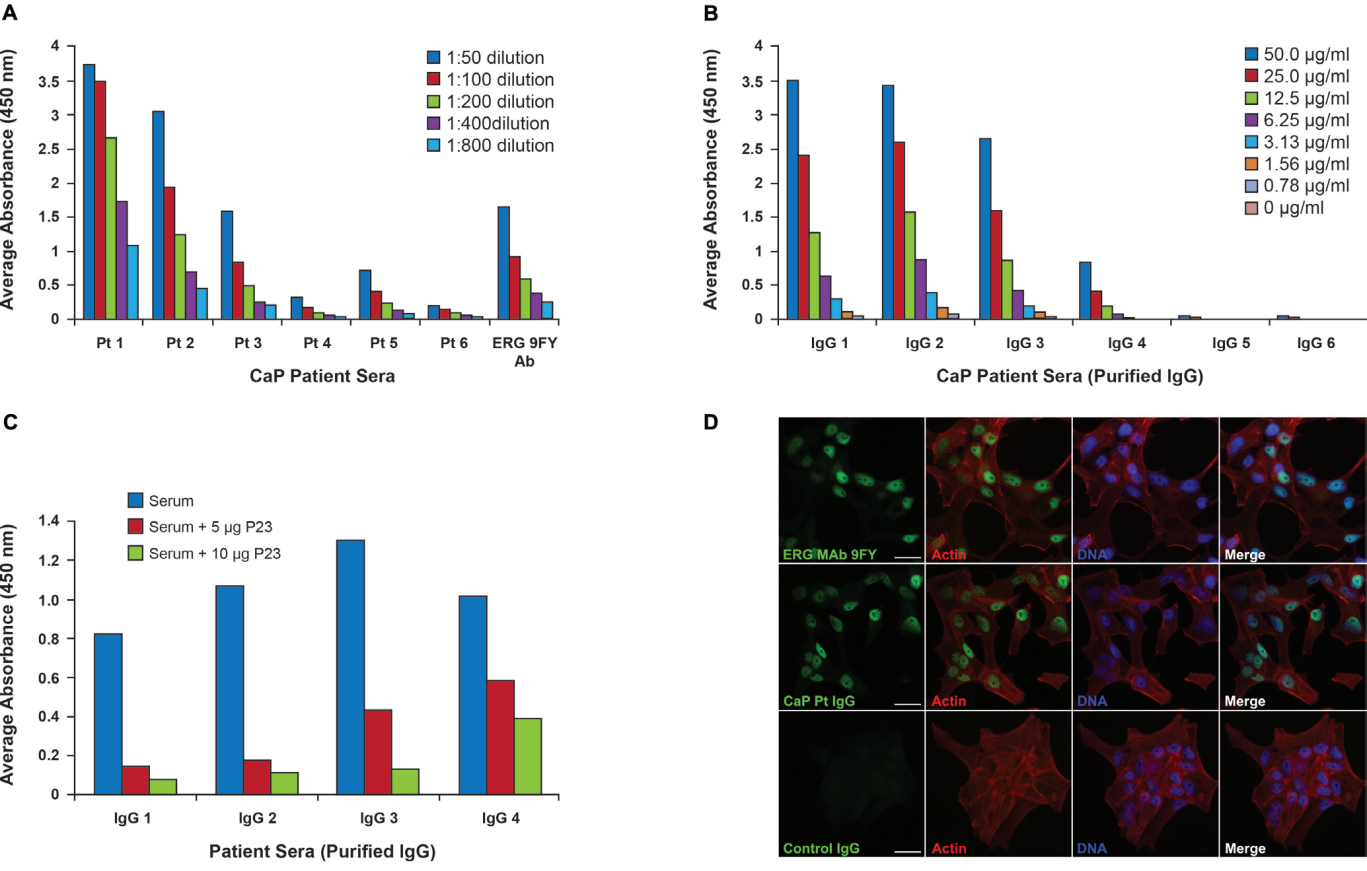
Specificity studies for ERG AAbs **A.** Analysis of AAb titers to ERG in selected CaP patients. Six candidate serum samples were serially diluted from 1:50 to 1:800 and assayed. Each dilution is represented by different color as shown. Results show that antibody titers decreased as dilutions increased, indicating specificity of the AAbs to the coated substrate; **B.** Specificity of total purified IgG from patient serum. Diluted IgG ranges from 50 μg/ml to 0 μg/ml and is represented by specific color as shown. Decreased reactivities of purified IgG upon serially dilution indicated specificity to ERG protein; **C.** Competition ELISA involving purified total IgG. Pre-incubation with P23 peptide, representing the N-terminal epitope of ERG, shows decreased reactivity with both 5 μg (red) and 10 μg (green) of peptide compared to sample alone (blue); **D.** Reactivities of purified IgG from CaP patients towards ERG protein expressed in VCaP cells by immunofluorescence assay. ERG MAb 9FY was used as a positive control. Green = ERG, panel 1; Red = Actin, panel 2; Blue = DAPI, panel 3; Merged image, panel 4. IgG Pt 4 represents CaP patient sera and IgG Pt 6 represents sera from healthy controls. Images taken at 400X; bar represents 25 μm.

In order to assess specificity of ERG AAbs to ERG protein, we evaluated dilutions of patient sera for reactivity. While the initial evaluation described in the previous section involved a dilution of 1:50 of the patient sera, we also carried out a detailed analysis involving multiple dilutions. Specifically, six candidate sera were selected from CaP patients (based on a range of AAb reactivity), which were further serially diluted and tested. The analysis of the sera by ELISA showed incremental reduction in absorbance values with dilution, which indicated ERG AAb specificity for the coated ERG protein. The ERG MAb 9FY was used as a positive control (Figure 3A).

### Serial dilution studies with purified immunoglobulin (IgG) from CaP patients positive by ELISA for reactivities towards ERG

Total IgGs were first purified from sera by spin columns as described in the methods. We selected six candidate sera consisting of 4 ERG AAb positive CaP patients and 2 healthy controls. Samples were serially diluted 1:2, starting at 50 μg. The results showed that purified IgGs from CaP patients exhibited absorbance values in accordance with the dilution of the sera (Figure 3B). The IgG from healthy controls showed no reactivity towards ERG. These data suggest that the reactivities noted are specific to ERG protein.

### Demonstration of the specificity of AAbs against ERG by competitive ELISA using purified IgG from the sera

The CPDR laboratory earlier identified an epitope at the N-terminal region of ERG protein based on studies with the ERG MAb 9FY [70]. The purified IgG, from the sera which were positive for reactivities towards recombinant ERG protein, also registered positive to a synthetic N-terminal peptide (corresponding to the identified epitope), designated P23. Competitive ELISA results revealed that purified IgG pre-incubated with the P23 peptide showed decreased absorbance in comparison to IgG by itself (Figure 3C). Further, pre-incubation with 5 μg and 10 μg of peptide with purified IgG from CaP patients showed a similar reactivity pattern. These results showed that the P23 peptide efficiently competed for purified IgG binding to the peptide coated on the microtiter wells, indicating specificity of the IgG to the ERG peptide.

### Specificity of the purified IgG towards ERG protein by immunofluorescence assay

To confirm the specificity of the AAbs to ERG, we have also utilized an immunofluorescence assay. VCaP cells are known to express ERG protein as they harbor the *TMPRSS2-ERG* gene fusion. These cells, grown on cover slips, were fixed and incubated with purified IgG from CaP patients or control sera followed by treatment with secondary antibody conjugate. As shown in Figure 3D, purified IgG from CaP patients recognized ERG protein in VCaP cells, similar to ERG MAb 9FY, which was used as a positive control. On the other hand, purified IgG from control sera did not bind to ERG in VCaP cells. F-actin and DAPI were used to visualize the cell structure and cell nucleus, respectively. These results independently and consistently validate the specificity of the AAbs to ERG protein.

### Demonstration of anti-ERG AAbs by luciferase immunoprecipitation systems assay

**Figure 4:**
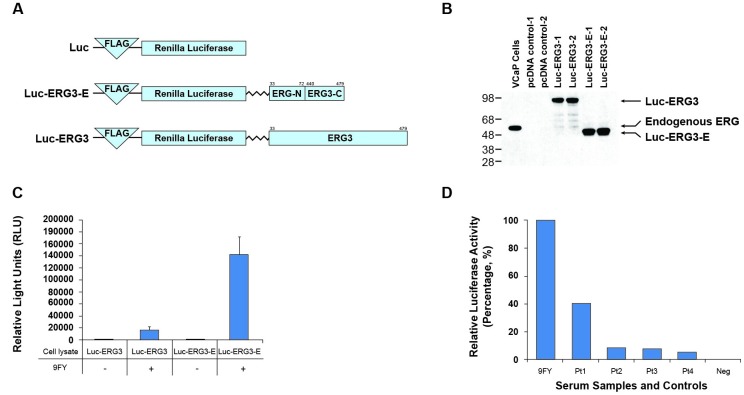
Detection of ERG AAbs in the sera by luciferase immunoprecipitation systems (LIPS) assay **A.** Schematic representation of recombinant DNA coding for chimeric luciferase-ERG constructs. Luc, backbone vector; Luc-ERG3, luciferase fused to full length ERG with a flexible linker; Luc-ERG3-E, luciferase fused to partial ERG with a flexible linker; **B.** Expression of chimeric luciferase-ERG protein in 293 cells. Chimeric proteins were probed by using 9FY antibody in an immunoblot assay; **C.** Analysis of Luc-ERG chimeric proteins as substrates for LIPS assay using ERG MAb 9FY; **D.** Analysis of ERG AAbs in patient sera by using chimeric Luc-ERG3-E protein.

To evaluate and reconfirm the presence of ERG AAbs in CaP patients, we also utilized another strategy, designated as a luciferase immunoprecipitation systems assay (LIPS). This assay is based on an enzymatic reaction and has been used to detect antibodies and AAbs in the human sera against pathogens and self-antigens, respectively [72]. The assay utilizes a chimeric protein, in which the tumor antigen is fused to luciferase enzyme coding sequences, as a substrate for capturing specific AAbs present in the sera. The luciferase activity in this assay is proportional to the amount of antibodies used for the reaction. The advantage with this assay is that it does not require purified recombinant protein for detecting antibodies. The schematic representation of the three recombinant plasmid constructs used for the assay is shown in Figure 4A. The first, designated Luc, contains FLAG epitope followed by luciferase coding sequences. Second, a Luc-ERG3 construct containing the FLAG epitope, luciferase, linker residues (10 amino acids) and the full length ERG. Finally, a Luc-ERG3-E, similar to Luc-ERG3, contains only epitopes from the N- and C-terminal regions (40 amino acids each) of the ERG protein. The expression of chimeric protein was verified in HEK293 cells by western blot, using ERG MAb 9FY (Figure 4B). The suitability of the chimeric proteins as substrates for capturing antibodies was first identified using ERG MAb 9FY. An aliquot of the cell extract was mixed with antibody (0.1 μg), pulled down by protein A/G beads, and processed for luciferase activity. Of the chimeric proteins, Luc-ERG3 showed less luciferase activity in comparison to the Luc-ERG3-E protein upon the addition of 9FY (Figure 4C), though both the proteins harbor the epitope for 9FY antibody. This suggests that the epitope recognized by ERG MAb 9FY is not equally accessible in the chimeric proteins, thus we have utilized Luc-ERG-E protein for further experiments. Cell extract (5 μl) from cells transfected with Luc-ERG3-E was used to test the patient sera. The results showed that AAbs against ERG can be detected in CaP patient sera (Figure 4D). The sera from healthy controls and CaP patients, negative for AAbs by ELISA, showed background level of luciferase activity. The sera positive for ERG AAbs by ELISA also registered positive in the LIPS assay.

### Anti-ERG AAbs recognize epitopes located at the N-and C-terminal regions of ERG protein

**Figure 5:**
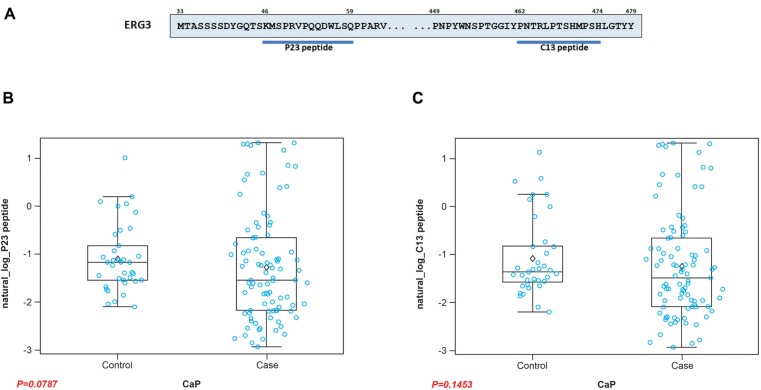
Analysis of selected ERG3 epitopes against ERG AAbs **A.** Schematic diagram of peptides corresponding to N- and C- terminal epitopes. Boxplots of the reactivities of patient sera against **B.** N- (P23) and **C.** C- (C13) terminal peptides as substrates. Results indicate that AAbs against both N-terminal and C-terminal epitopes of ERG3 are found in patient serum.

The humoral response in a patient comprises antibodies against a number of epitopes present on a protein. In accordance with this, we tested the reactivity of the serum AAbs against different epitopes of ERG. Previously our laboratory showed that the N-terminal P23 peptide, comprising the residues “46-KMSPRVPQQDWLSQ-59”, binds to ERG MAb 9FY with an affinity similar to the full length ERG protein [70]. Similarly, a C-terminal peptide, designated C13, containing the residues “462-PNTRLPTSHMPSH-474” (Figure 5A), was recognized by the Epitomics rabbit MAb (unpublished data). Both peptides are unique to ERG protein based on BLAST analysis. Analysis of the CaP patient sera using N- and C-terminal peptides as substrates showed values of *p* = 0.0787 and *p* = 0.1453, respectively (Figure 5B, 5C), indicating that these epitopes are recognized by the host immune system for generating AAbs. The results also indicate that the extent of reactivities of the patient sera against peptide substrates was lower likely due to binding of AAbs to only one epitope in comparison to multiple epitopes present on the full length ERG protein. Further, these results also suggest that sera from several CaP patients may not harbor AAbs against both N- and C-terminal epitopes.

### Identification of ERG isoform specific AAbs in the sera of CaP patients

**Figure 6:**
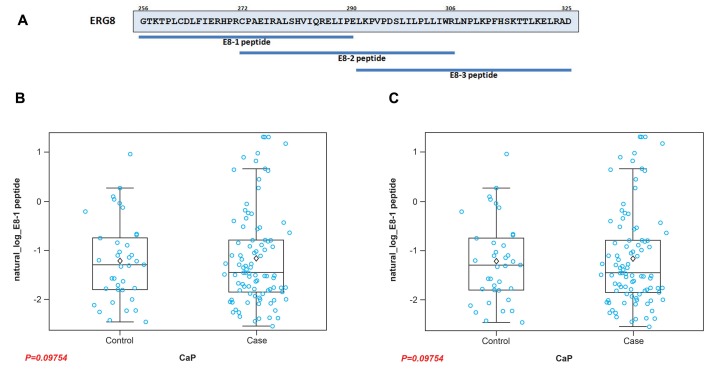
Peptides representing unique sequence of the ERG8 isoform **A.** Schematic diagram of ERG8 peptides used as a representative of the ERG Type II splice variant. Reactivities of the patient sera against Type II isoform specific peptides (E8-1 and E8-2) are shown as Box plots **B**., **C**., respectively.

The analysis of CaP associated fusion transcripts has revealed the presence of multiple splice variants, potentially exhibiting different biological activities and correlating with different tumor phenotypes [73-77]. These variants can be divided into near full length (lacking 32 N-terminal amino acids) Type I ERG, containing the DNA-binding domain (DBD), and Type II ERG, a truncated form lacking DBD coding sequences [74]. The ratio of these variants has also been found to be associated with *MYC* oncogene expression and biochemical recurrence [78, 79]. Therefore, it was of interest to raise the question as to whether isoform specific AAbs against ERG exist in the sera of CaP patients. To address this, we selected two transcript variants: ERG3 (as a representative of Type I transcript variant) and ERG8 (as a representative of Type II transcript variant) for analysis. To enable the detection of AAbs unique to each isoform, we used synthetic peptides corresponding to epitopes present in the respective variant. While the C13 peptide (Figure 5A) is specific to isoforms encoded by Type I splice variants, in order to detect ERG8 specific AAbs, we synthesized three peptides (E8-1, E8-2 and E8-3) based on the unique amino acid sequence present at the C-terminus of ERG8 for analysis (Figure 6A). Of the three peptides, E8-3 was found to be weakly immunogenic in the initial analysis, therefore it was not included for assessing the reactivities of CaP patient sera (data not shown). On the other hand, E8-1 and E8-2 peptides were recognized by patient sera with values of *p* = 0.9754 and *p* = 0.0454, respectively, by ELISA. The results provide support that isoform specific AAbs are also present in CaP patient sera (Figure 6B, 6C). As noted with N- and C-terminal peptides in the previous section, ERG8 specific peptides showed similar reactivities with AAbs in the sera. These data lend support to the view that unique ERG8 segment is likely less immunogenic in nature.

### Utilization of a panel of AAbs as biomarkers for the diagnosis of CaP

**Figure 7:**
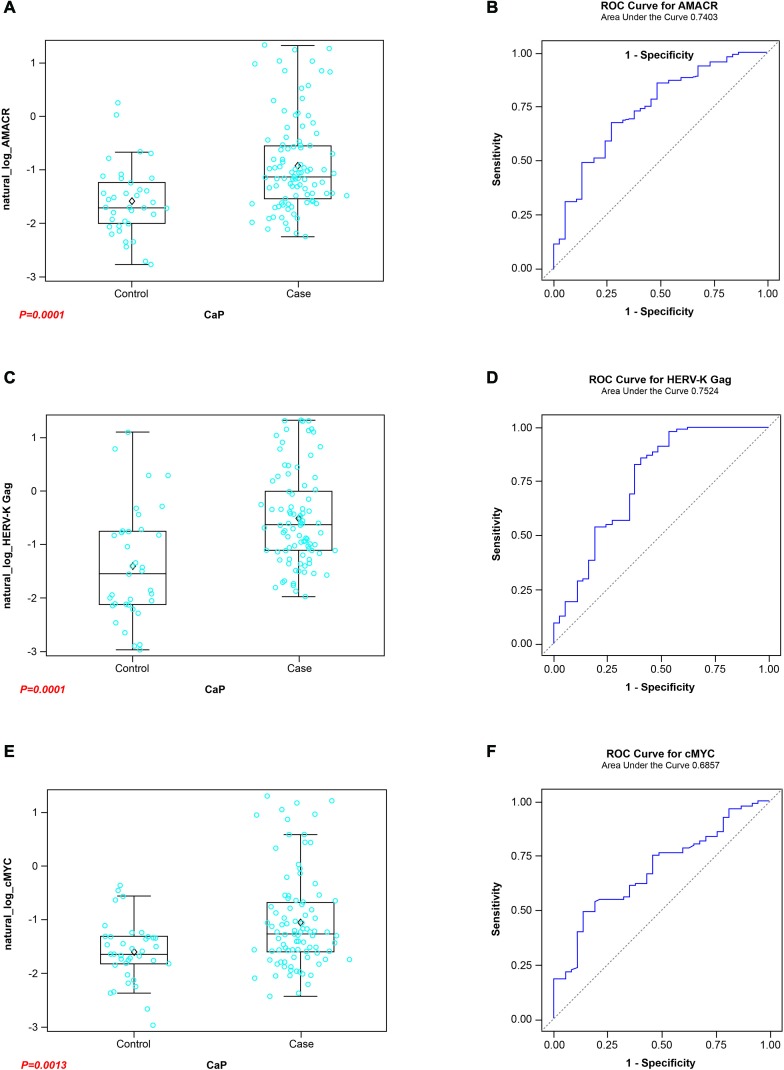
Boxplots of the reactivities of the AAbs to A Full length AMACR protein, (C) HERV-K Gag derived peptide, and (E) Full length C-MYC protein in the sera of CaP Cases and Healthy Control groups. ROC curves for AMACR, HERV-K Gag, and C-MYC are shown in B, D, and F, respectively.

**Figure 8:**
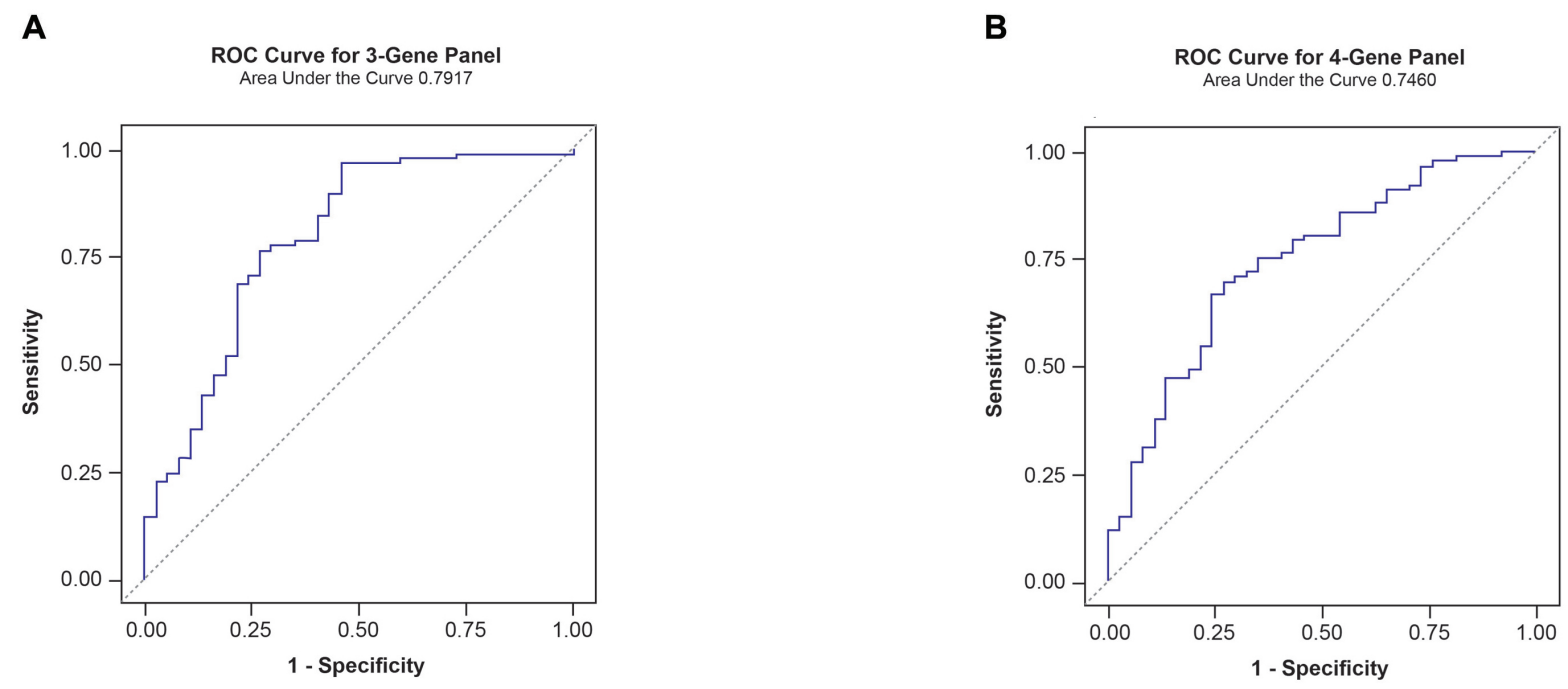
Receiver operator characteristic analysis of a panel of AAbs **A.** ROC curve for a 3-gene panel of AAbs comprised of ERG, AMACR, and HERV-K Gag (AUC=0.792). **B.** ROC curve for a 4-gene panel of AAbs comprised of ERG, AMACR, HERV-K Gag, and C-MYC (AUC=0.746).

As discussed previously, a single analyte such as anti-ERG AAbs is likely to show positivity only in a subset of CaP patients. Given that CA CaP patients harbor *TMPRSS2-ERG* only in an estimated 50% of cases, it is probable that additional genes may contribute to the development of CaP. In addition, it has been shown that AAbs against a single antigen show low sensitivity and a high specificity [29]. However, an approach utilizing a combination of AAbs or a multiplex AAbs combined with other tumor markers such as antigen has shown an enhanced performance in sensitivity [29]. For this reason, we have considered several genes, based on the expression and/or AAb analysis by investigators, for inclusion in a panel. Specifically, AMACR and endogenous HERV-K Gag were included in this study panel as AAbs against both have been noted in CaP patients [80-84]. While full length protein was used as a substrate for AMACR, a synthetic peptide containing residues “243-YPQPPTVRLNPTASRSGQGG-262” was used for HERV-K Gag in this study. In addition, C-MYC was added for the following reasons: i) C-MYC overexpression has been noted in CaP previously [78, 85-87]; ii) AAbs against C-MYC have been noted in breast and other cancers [88]; iii) Analysis of VCaP cells, charcoal stripping followed by R1881 treatment, showed a close correlation between ERG and C-MYC [79]. Upon analysis of the sera by ELISA, it was noted that the reactivities from CaP patients were significant compared to healthy controls for AMACR, HERV-K Gag, and C-MYC, which registered values *p* < 0.0001, *p* < 0.0001 and *p* = 0.0013, respectively. To evaluate the performance of individual AAbs as biomarkers for the detection of CaP, an ROC curve analysis was performed. The results showed an AUC of 0.740, 0.752 and 0.685 against AMACR, HERV-K Gag, and C-MYC, respectively (Figure 7). In addition, correlations were examined among the genes selected in this study. The results, shown in Table 2, indicate a significant correlation between ERG and HERV-K Gag and between AMACR and C-MYC. Subsequently, in order to evaluate the performance of a combination of AAbs as biomarkers for the detection of CaP, an ROC curve analysis was conducted, using a combination of ERG, AMACR and HERV-K Gag as a 3-gene panel, which showed a value of AUC = 0.792. In addition, a combination of ERG, AMACR, HERV-K Gag, and C-MYC was evaluated as a 4-gene panel (AUC 0.746) (Figure 8).

**Table 2:** Correlation between genes

Gene 1	Gene 2	R	P-value
Natural log of ERG3	Natural log of AMACR	0.64	< 0.0001
	Natural log of c-MYC	0.72	< 0.0001
	Natural log of GAG	0.87	< 0.0001
Natural log of AMACR	Natural log of c-MYC	0.87	< 0.0001
	Natural log of GAG	0.67	< 0.0001
Natural log of c-MYC	Natural log of GAG	0.72	< 0.0001

### Evaluation of ERG AAbs in an independent cohort of CaP patients

**Figure 9:**
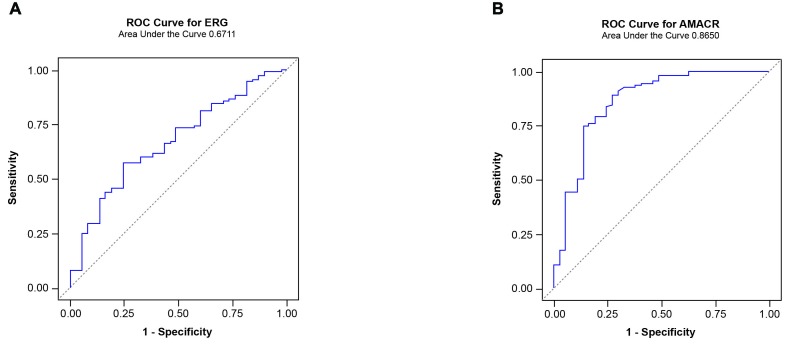
Receiver operator characteristic analysis of AAbs in an independent cohort of CaP patients **A.** ROC curve for AAbs against ERG. **B.** ROC curve for AAbs against AMACR.

An independent cohort was also tested for ERG AAbs which was comprised of 117 CA CaP patients. The distribution of patients according to Gleason was the following: Gleason 6 or less, *n* = 32; 7 (3+4), *n* = 28; 7 (4+3), *n* = 28; 8-10, *n* = 29. The results showed that ERG AAbs were found to be significantly higher in CaP cases than healthy controls (*p* = 0.0022). The pattern of AAb reactivity observed towards ERG in this cohort is similar to that of the previous cohort. The reactivity of AAbs towards AMACR also showed a significant value (*p* < 0.0001). Receiver operating characteristic curve analysis registered an AUC value of 0.6711 and 0.8650 for ERG and AMACR, respectively (Figure 9). The data indicate that AAbs against ERG and AMACR are present in additional cohorts of CaP patients. We are currently in the process of assembling sera from biopsy negative and positive for CaP for evaluation of the sensitivity and specificity of our panel for CaP detection.

## DISCUSSION

Screening methods for the diagnosis and prognosis of cancer have played a critical role in the management of cancer. These comprise both invasive (biopsy) and non-invasive or minimally invasive approaches. In comparison to assays using invasive approaches, a liquid biopsy – an assay based on the analysis of biomarkers in body fluids such as blood, sera, or plasma – would be of high value for early and rapid detection and diagnosis of cancers, including CaP. The advantages with such assays are that the results can be generated within a short time, and are cost effective. In this regard, AAbs against tumor antigens are ideal biomarkers that can be exploited for both diagnosis and prognosis of cancer. The detection of AAbs in patients well before the onset of symptoms of breast cancer and lung cancer [20, 89] suggests the possibility that AAbs may appear early on, and present themselves sooner than would be detected in a slow growing cancer. The physiological role of AAbs in cancer is not clear. The conventional paradigm suggests that immune responses in the form of AAbs, as well as cellular immune responses, are elicited to interfere with the initiation and progression of disease [19]. In support of this, it was reported that antibodies against glucose-regulated protein 78 from CaP patient serum have been shown to modulate cell proliferation in vitro [90].

While numerous AAbs are present in human sera [91], it has been demonstrated that distinct profiles of AAbs are associated with specific diseases including Alzheimer's disease, Parkinson's disease, and cancer [91-93]. AAbs against several TAAs have been demonstrated in many different cancers, including CaP [24, 94-99]. In a scenario where there is no information available regarding analytes, the identification of candidate self antigens for AAbs has been performed by several methods including SEREX, SERPA, MAPPing, and phage display, in combination with the use of sera from cancer patients [25]. A positive signal leads to the identification of a protein which is then used for characterization of AAbs in patients. Alternatively, when there is information available regarding the expression status of genes and/or proteins, we have the option of selecting specific candidate protein as a substrate for exploring the detection of AAbs. In the context of CaP, we utilized the latter approach, as overexpression of ERG has been previously reported from analysis of patient specimens [52, 56, 60, 62, 66]. Despite the documentation of the *TMPRSS2-ERG* gene fusion in about 50% prostate CA cancer patients, there is no information available regarding the presence of AAbs against ERG protein. This was the basis for our analysis of AAbs against ERG in CaP patient serum.

The novel finding presented here is that ERG oncoprotein elicits B cell immune responses in CaP patients. Kissick et al. [100] reported earlier on ERG specific CTLs noted in CaP patients. Interestingly, ERG is an intracellular protein. Hence, it is likely that ERG protein may be released from tumor cells by necrosis, cell lysis, or micro-vesicle shedding which is then recognized by immune system. Analysis was carried out using sera from a cohort of 130 individuals comprised of 93 CA CaP patients and 37 sera from age matched CA healthy control subjects. Our studies showed, for the first time, that ERG is a target for the generation of AAbs. The extent of seropositivity varies between CaP patients. It is likely that multiple factors may contribute to our observations, including level of ERG expression in primary tumors, immune surveillance of the host, tumor heterogeneity, MHC background and antigen presentation. However, the presence of anti-ERG AAbs was found to be lower in the sera from normal healthy individuals.

Studies reported in the past 20-30 years have indicated that the host immune system, in addition to recognizing the exogenous proteins of viral, bacterial, and parasitic origin, can also recognize self proteins [19]. However, the recognition by the immune system with respect to the latter category is based on changes or alterations in the self proteins. These changes may include overexpression, mutation, glycosylation, phosphorylation, and misfolded proteins. In the context of CaP, a notable observation that was reported a decade ago was genetic rearrangements leading to the generation of fusion genes. An intrachromosomal deletion resulted in the generation of a predominant fusion gene in which *ERG* coding sequences are linked to the androgen receptor regulated promoter region of the *TMPRSS2* gene (*TMPRSS2-ERG*). The biological consequence of this gene fusion is the overexpression of ERG protein. Interestingly, the expression of ERG protein is completely absent in prostate tissues under normal conditions [62]. This scenario prompted us to hypothesize that ERG overexpression may lead to the induction of anti-ERG AAbs, which may in turn serve as a biomarker for detecting CaP. Our studies, indeed, provide evidence in support of the presence of ERG-AAbs in the sera of CaP patients.

The specificity of AAbs against ERG protein was evaluated, as this would be a pre-requisite for their use in the diagnosis/prognosis of CaP. We have utilized multiple approaches including serial dilution of patient sera and purified total IgG, competition assay involving peptides representing an epitope in the ERG protein, and staining of cells expressing ERG protein with purified IgG from patient sera. In addition, the humoral immune response generally comprises both continuous (linear) and discontinuous epitopes. Accordingly, peptide epitopes derived from the N- and C-terminal regions of ERG also showed reactivities in the sera, indicating that AAbs target distinct epitopes in the protein. The demonstration of anti-ERG AAbs is not completely surprising. Studies published on AAbs have shown that antigens responsible for the generation of AAbs belong to cell cycle, signal transduction, mRNA transport, proliferation, and apoptosis pathways [19]. ERG has also been shown to have an active role in differentiation, as inhibition of ERG expression through siRNA in VCaP cells leads to the differentiation of cells [78].

Studies by investigators have already identified several antigens as the source for generation of AAbs in CaP. These include NY-ESO-1, XAGE1b, SSX-2 and 4, AMACR, p90, LEDGF, TARDBP, TLN1, PARK7, CALD1, TTLL12, p62, Koc, Cyclin B1, PKACA, HIP1 and Survivin, MUT, RAB11B, CSRP2, SPOP, RalA and ZNF671 [30, 95, 97, 99, 101, 102]. Recently, several groups have reported the presence of AAbs against endogenous retrovirus Gag protein [83, 84] and also transcripts in CaP cells [81]. Our data presented here add ERG to this list of antigens. ERG AAbs may be of value in both diagnosis and prognosis of CaP for the following reasons: i) ERG expression level is high in 30-50 % of CaP patients of diverse ethnic groups; ii) ERG expression is also implicated as a prognostic biomarker although this needs to be further evaluated [103]. In an effort to enhance the diagnostic sensitivity of autoantibodies, a panel approach was considered, as has been shown in lung cancer [88, 89, 104]. In our study, the AAb panel comprising ERG, AMACR, and HERV-K Gag yielded an AUC of 0.792 for differentiating cancer cases from healthy controls. It is likely that the combination of AAbs may improve the efficiency of the diagnostic test through additivity.

As ERG is a member of the ETS family of proteins, it is likely that AAbs to ERG may not be specific to only ERG. This raises the question whether ERG AAbs target evolutionarily conserved domains of the ETS family members. The ERG related members include ETV1, ETV4, ETV5 and FLI1 which have been shown to overexpress in different diseases including CaP, Ewing sarcoma, and acute myeloid leukemia [105-107]. This situation warrants the determination of immunoreactive residues of the ERG protein for AAbs through overlap peptide scanning approach. Our studies highlight the possibility that testing for AAbs in other diseases may also benefit patients.

A proper understanding of the clinical relevance of the anti-ERG AAbs detected in CaP patients is critical because of the evidence that antibodies may precede the clinical onset of disease by many years. There is a possibility that ERG AAbs may reflect a change in the tumor stage or treatment. In a recent review, Zaenker et al. [93] noted that high titers of AAbs have been associated with regulatory T cell downregulation. This situation may lead to activation of effector T cells and antibody producing plasma cells, which can impact tumor growth. Based on this, it is tempting to suggest that anti-ERG AAbs may exert their effect by acting on multiple proteins either through transcriptional modulation and/or protein-protein interactions. Hence, it is important to assess whether a positive correlation exists between anti-ERG AAbs and disease progression/survival of CaP patients. Previous studies from our laboratory showed that a high Type I/Type II ratio of *ERG* gene transcripts correlated with poor prognosis, and a low ratio was associated with favorable clinical-pathologic data [74, 79] based on RT-PCR using tumor cells. A novel finding from our study is that ERG isoform specific AAbs were also noted in patient sera, utilizing peptides unique to each transcript variant encoded protein. Considering this, we reasoned that it would be relatively easy to quantify ERG isoforms using an assay based on markers such as AAbs, in comparison to RT-PCR. Hence, the use of isoform specific AAbs as prognostic indicators for CaP is appealing. Overall, the data presented in this study demonstrated the presence of AAbs against ERG oncoprotein in the sera of patients with CaP, which may aid in the early detection of CaP. In addition to diagnosis, ERG may also serve as a candidate antigen for developing immunotherapies against CaP. It was also shown that a combination of AAbs have clinical relevance for the detection of individuals with CaP over controls. The prevalence of anti-ERG AAbs represents a potentially important biomarker that can not only be used to stratify CaP patients but also predict the potential for biochemical recurrence or metastatic disease.

## MATERIALS AND METHODS

### Procurement of samples

The patient serum samples used in this study were obtained before radical prostatectomy procedures under an IRB-approved protocol (No. 390559) at Walter Reed National Military Medical Center with written consent. For CaP cases, blood was collected at the time of surgery. Serum was separated and stored at −80°C until use. Samples were heat inactivated at 55°C for 30 minutes before use in ELISA experiments. Upon heat-inactivation, samples were stored at 4°C, and used within 4-6 weeks of inactivation. The sera derived from two independent cohort of CaP patients were used for analysis. The training cohort consisted a total of 130 with 93 CaP cases, and 37 healthy controls of CA origin. Control cases were healthy males who had PSA levels ≤ 2 ng/ml. An additional cohort of 112 CA patients representing different Gleason grades was also included for ERG AAb evaluation.

### Recombinant proteins and peptides

Recombinant full length ERG protein, produced in mammalian cells upon transfection of expression plasmid DNA, was purchased from Origene (Rockville, MD). Recombinant proteins for AMACR and cMYC, produced in mammalian cells, were also purchased from Origene. Peptides representing ERG3, ERG8, and HERV-K Gag epitopes were synthesized by a commercial vendor (Infinity Biotech, PA).

### AAb detection by enzyme-linked immunosorbent assay (ELISA)

ELISA procedures were carried out in NUNC 96-well flat bottom Maxisorp plates (Thermo Scientific; Rockford, IL). Plates were coated with 50 ng/well protein, or 500 ng/well peptide, using 100 μl Coating Buffer (50 mM NaHCO3, pH 9.6). The plates were covered with microplate sealers (Pierce/Thermo Scientific, Rockford, IL) and incubated at 4°C overnight. The next day, plates were washed 4 times with wash buffer (1X PBS+Tween20, KD Medical; Columbia, MD) and blocked with 200 μl blocking buffer (StartingBlock; Thermo Scientific), covered, and incubated for 1 hour at room temperature (RT). Inactivated serum samples were diluted 1:50 in Dilsim II buffer. After blocking, plates were washed once with wash buffer, and incubated with 100 μl diluted serum samples, covered for 1 hour at 37°C. All reactions were carried out in duplicate. Plates were again washed 4 times with wash buffer and then incubated with 100 μl of an HRP-conjugated anti-human antibody (KPL Inc.; Gaithersburg, MD), diluted 1:60,000 in ELISA diluent (20% NGS in 1X PBS with 0.1% Triton-X 100), covered for 1 hour at 37°C. Plates were washed 4 times with wash buffer and 100 μl of K-Blue Aqueous TMB substrate (Neogen; Lexington, KY) was added to the plates, and incubated uncovered for 30 min at RT. Sulphuric acid (100 μl of 2N) was added to the plates post-incubation to stop the reaction. Plates were immediately read at 450 nm to measure absorbance.

### Spike-in experiments

Microtiter plates were coated with 50 ng/well of ERG protein. Six candidate sera for patients, which were negative for ERG AAbs, were used for spike-in experiments. Serum samples were diluted 1:50 in Dilsim II dilution buffer, and spiked with 10 ng/mL ERG 9FY mouse MAb. Samples were loaded into microtiter wells in duplicate and assayed as above. An HRP-conjugated anti-mouse antibody (KPL Inc.; Gaithersburg, MD), diluted 1:20,000, was used for detection.

### Specificity assays

We conducted a series of assays to examine specificity of ERG AAbs to ERG protein coated on microtiter plates: i) Six candidate serum samples were first diluted 1:50 as before and then further serially diluted 1:2 to obtain a final dilution of 1:800. Dilutions of each sample were loaded onto microtiter plates and assayed as written above. ii) Six candidate serum samples were selected including 4 from CaP patients, and 2 from healthy controls. Total IgG was first purified using NAb™ Protein A Plus Spin Columns, and then desalted using Zeba™ Spin Desalting Columns. The final concentration of IgG was measured using a Nanodrop spectrophotometer. Total IgG for each sample was diluted to a starting concentration of 50 μg/ml and then serially diluted 1:2 to reach a final concentration of 0.78 μg/ml. Dilutions of each sample were loaded onto microtiter plates in duplicate and assayed as written above. iii) For competition studies, the purified IgGs were first pre-incubated with the P23 peptide corresponding to the ERG MAb 9FY epitope (5 μg and 10 μg) for 30 minutes at RT, and then added to microtiter plates. The samples were then assayed in duplicate as written above.

### Antigen detection ELISA

The methodology used for ERG antigen detection is as described in [71]. Briefly, NUNC 96-well flat bottom Maxisorp plates (Thermo Scientific, Rockford, IL) were coated with 1 μg/mL of ERG MAb 9FY, using 100 μL coating buffer (50 mM NaHCO3, pH 9.6). The plates were covered with microplate sealers (Pierce/Thermo Scientific, Rockford, IL) and incubated at 4°C overnight. The next day, plates were washed 4 times with wash buffer (1X PBS +Tween-20; KD Medical, Columbia, MD) and blocked with 200 μL blocking buffer (StartingBlock; Thermo Scientific), covered, and incubated for 1 hour at room temperature (RT). Inactivated serum samples were diluted 1:50 in Dilsim II buffer. After blocking, plates were washed once with wash buffer, and incubated with 100 μl diluted serum samples, covered for 1 hour at 37°C. All reactions were carried out in duplicate. Plates were again washed 4 times with wash buffer and then incubated with 100 μL biotinylated ERG antibody (2 μg/mL; Origene), covered for 1 hour at 37°C. Plates were washed 4 times with wash buffer and then incubated with 100 μL of Streptavidin-HRP conjugated antibody (KPL Inc., Gaithersburg, MD), diluted 1:10,000 in ELISA diluent (20% NGS in 1X PBS with 0.1% Triton-X 100), covered for 1 hour at 37°C. After incubation, plates were washed 4 times with wash buffer, and 100 μL of K-Blue Aqueous TMB substrate (Neogen, Lexington, KY) was added to the plates, then incubated uncovered for 30 min at RT. Sulfuric acid (2 N, 100 μL) was added to the plates post-incubation to stop the reaction. Plates were immediately read at 450 nm to measure absorbance.

### Immunofluorescence assay

VCaP cells were cultured on poly-L-lysine-coated glass coverslips (BD Bioscience) in androgen-deprived medium for 2 days. Cells were induced with 0.1 nmol/L R1881 and cultured for another 48 hours. Cells were fixed with 4% paraformaldehdye buffered in PBS, permeabilized with 0.1% Triton X-100 in PBS, and blocked with 1% normal horse serum (Vector Laboratories) before incubating with purified IgG. Secondary antibody (Alexa Fluor-488 goat anti-human; Invitrogen) was subsequently applied together with DAPI (40, 6-diamidino-2-phenylindole). F-actin was stained with Alexa Fluor-594 phalloidin (Invitrogen). ERG MAb 9FY was used as a positive control and stained with Alexa Fluor-488 goat anti-mouse secondary antibody. Images were captured using a 40x/0.65 N-Plan objective on a Leica DMIRE2 upright microscope with a QImaging Retiga-EX CCD camera controlled by OpenLab software (PerkinElmer), converted into color, and merged by using Adobe Photoshop.

### Luciferase Immunoprecipitation Systems (LIPS) assay

Renilla Luciferase constructs (Luc-ERG3-E, Luc-ERG3) were generated in pcDNA3 through a commercial vendor (GenScript, Piscataway, NJ, USA). The Luc-ERG3-E construct contained Renilla Luciferase coding sequences (AF416990.1) linked to a 40 amino acid N-terminal ERG3 sequence (residues 33-72) followed immediately by a 40 amino acid sequence at the C-terminus of ERG3, while the Luc-ERG3 contained the entire 447 amino acid sequence of ERG3. The methodology for the LIPS assay was followed from Burbelo et al. [72]. Briefly, HEK293 cells were transfected with Luc-ERG3-E, Luc-ERG3, or pcDNA3 empty vector 48 hours after plating in 100 mm^2^ dishes. Forty-eight hours post-transfection, the plates were washed twice with PBS, and scraped with 1 ml of Lysis Buffer (20 mM Tris, pH 7.5, 150 mM NaCl, 5 mM MgCl2, 1% Triton X-100) plus 50% glycerol and protease inhibitors. Once collected, lysates were sonicated, centrifuged at 13,000 × g for 4 min, supernatants collected and used immediately or stored at −20°C. Total luciferase activity was measured in crude extract by adding 1 μl lysate to 100 μl of assay buffer (Dual Luciferase Reporter Assay System, Promega, Madison, WI, USA) immediately measuring light-forming units with a luminometer (Victor3). The expression of ERG3 protein in the cell extract was verified by western blot using ERG MAb 9FY.

Immunoprecipitation assays were performed in 100 μl volumes containing 6 μl of a 50% suspension of protein A/G beads (in PBS), 10 μg of purified total IgG or 5 μl of sera, 10 μl of HEK293 cell extract, and Lysis Buffer. The suspension was incubated at 4°C with rotation for 2 hours, washed 4 times with 1 ml of cold Lysis Buffer and one time with 1 ml of PBS. After the final wash, the beads, in a volume of about 10 μl, were loaded onto Blackwell 96-well plates and evaluated for luciferase activity using the Dual Luciferase assay kit as described above.

Cells were lysed in situ 24 hours post-transfection, rocked for 15 minutes at room temperature, and centrifuged at 15,000 x g for 15 minutes to pellet the cell debris. Cell supernatants were loaded onto Blackwell 96-well plates and evaluated for luciferase activity using the Dual Luciferase Reporter Assay System (Promega, Madison, WI, USA).

### Western blot

HEK293 cells were grown in 100 mm^2^ dishes and transfected at 50% confluence with 5 μg of each Luc construct. 48 hours post-transfection, cell pellets from transfection procedures were lysed in Mammalian Protein Extract Reagent (M-PER; Pierce/Thermo Scientific, Rockford, IL, USA). Following pre-cleaning by centrifugation, protein concentrations of cell lysates were determined by using Protein Assay Reagent (Bio-Rad, Hercules, CA, USA). Lysates equivalent to 25 μg of protein were separated on NuPAGE Bis-Tris (4-12%) gels (Invitrogen, Carlsbad, CA, USA) and transferred onto PVDF membranes. Membranes were blocked in Blocking Buffer (LI-COR, Lincoln, NE, USA) and incubated with the ERG MAb 9FY (Biocare Medical Inc., Concord, CA, USA). Membranes were washed in Tris-Buffered Saline + Tween 20 (TBST) before incubation with sheep anti-mouse secondary antibodies (GE Life Sciences, Pittsburgh, PA, USA).

### Statistical analysis

Demographic and clinical characteristics were compared between case and control groups using the Wilconson rank sum test (age, serum PSA); Pearson correlation analysis was used to evaluate the correlation among four gene AAb (log-transformed); boxplot and Wilconson rank sum tests were used to examine the difference of four gene AAb levels (log-transformed) for case versus controls.

Univariable logistic regression and ROC curve analysis were used to examine the CaP prediction value of four gene AAb levels. Considering the strong correlations among those four gene AAb levels, principal component analysis (PCA) was used to generate summary variables of those four AAb levels, which was used in the four gene combination panel ROC analysis.

All statistical analyses were performed using SAS version 9.3 (SAS Institute, Cary, NC). All P-values were computed using two-sided statistical tests (summary alpha = 0.05).
